# Chitosan Nanofiber and Cellulose Nanofiber Blended Composite Applicable for Active Food Packaging

**DOI:** 10.3390/nano10091752

**Published:** 2020-09-04

**Authors:** Le Van Hai, Lindong Zhai, Hyun Chan Kim, Pooja S. Panicker, Duc Hoa Pham, Jaehwan Kim

**Affiliations:** 1 CRC for Nanocellulose Future Composites, Inha University, Incheon 22212, Korea; levanhai121978@gmail.com (L.V.H.); duicaofei@naver.com (L.Z.); kim_hyunchan@naver.com (H.C.K.); pooja.panicker7@gmail.com (P.S.P.); phamduchoa.tdt@gmail.com (D.H.P.); 2 Pulp and Paper Technology Department, Phutho College of Industry and Trade, Phutho 290000, Vietnam

**Keywords:** cellulose nanofiber, chitosan nanofiber, composite, mechanical properties, antioxidant activity

## Abstract

This paper reports that, by simply blending two heterogeneous polysaccharide nanofibers, namely chitosan nanofiber (ChNF) and cellulose nanofiber (CNF), a ChNF–CNF composite was prepared, which exhibited improved mechanical properties and antioxidant activity. ChNF was isolated using the aqueous counter collision (ACC) method, while CNF was isolated using the combination of TEMPO oxidation and the ACC method, which resulted in smaller size of CNF than that of ChNF. The prepared composite was characterized in terms of morphologies, FT-IR, UV visible, thermal stability, mechanical properties, hygroscopic behaviors, and antioxidant activity. The composite was flexible enough to be bent without cracking. Better UV-light protection was shown at higher content of ChNF in the composite. The high ChNF content showed the highest antioxidant activity in the composite. It is the first time that a simple combination of ChNF–CNF composites fabrication showed good mechanical properties and antioxidant activities. In this study, the reinforcement effect of the composite was addressed. The ChNF–CNF composite is promising for active food packaging application.

## 1. Introduction

Nowadays, the use of renewable materials instead of plastics is essential since the proliferation of plastics in the environment has been known to create various health and ecological problems. An example of extensive use of plastics is packaging. Recently, packaging was identified as an essential element to address the key challenge of sustainable food consumption [1]. When a food product is thrown away, the packaging is also discarded, leading to an additional environmental burden. Thus, petroleum-based packaging materials need to be replaced with renewable materials.

Chitosan, the second most abundant material after cellulose on Earth, is renewable, biodegradable, biocompatible, non-toxic and capable of transporting antioxidants [2,3,4]. Chitosan has been investigated for various applications including drug delivery, artificial skin, wound-dressing and biomedical and pharmaceutical applications [5]; contact lens and, water filtration [6]; food packaging [7,8,9]; and fruit preservations such as tomatoes, carrots and raw shrimps [10,11,12,13]. Chitosan does not cause any intrinsic food contamination such as phthalate leaching, thus has emerged as suitable alternatives for commercial plastics. Chitosan can be formed as a nanofiber. Chitosan nanofiber (ChNF) is beneficial to various applications since it has high aspect ratio, good chemical/physical interaction and flexibility. Most ChNFs can be fabricated by electro-spinning process including dissolving and purifying steps [14]. Instead of dissolving, ChNF can be isolated from its raw materials by physical methods, for example, by using supermasscolloider and high water jet pressure, above 200 MPa [15,16]. ChNF is considered for many applications such as removal of Arsenate [17], biomedical applications [18,19] and filtration membranes [14,20].

Cellulose, the most abundant polymer on Earth, has been used for long time. However, it has become a very interesting subject in recent years due to its possibility for substituting petroleum-based materials, and it is readily available around the world. Cellulose has been used for a wide variety of applications such as paper, packaging, composites, textiles, biomedical and pharmaceutical applications [21,22]. Cellulose nanofiber (CNF) is a nano-sized fiber in the range of ten to a couple hundred nanometers. Since CNF has merits over cellulose nanocrystals, its market is remarkably increasing for various applications [23]. It can be prepared mainly by mechanical and chemical methods. 2,2,6,6-tetramethylpiperidine-1-oxylradical-oxidation (TEMPO-oxidation) is a chemical method to extract CNF from various cellulose resources [24,25].

Cellulose and chitosan have been studied for food packaging materials [3,4,21,22]. It is well-known that chitosan has antibacterial, antioxidant and good food preservation properties. Cellulose has also been used as a food packaging material for long time. Early studies explored cellulose–chitosan composites for food packaging materials [26,27]. However, the blending of CNFs and ChNF has not been employed in any advance research, which could be applicable for food packaging. Thus, in this research, two types of nanofibers, namely CNF and ChNF, were blended for a potential active food packaging material. IThe ChNF was isolated using a physical treatment, so-called, aqueous counter collision (ACC) method [28]. CNF was also prepared from softwood pulp using a combination of chemical method, TEMPO-oxidation, and the ACC method to further decrease its size. We intended to distinguish the ChNF and CNF size to blend them with different morphologies. The prepared ChNF and CNF were directly blended to prepare ChNF–CNF composites. The advantages of blending CNF and ChNF are simple and benign preparation. Furthermore, by distributing various sizes of ChNF and CNF, physical and functional properties of the composite can be controlled. Active food packaging or smart packaging for food products refers to packaging that has functionalities in protecting the products. Those functionalities include preserving freshness and antimicrobial activity. Previous studies have reported that chitosan exhibits the functionalities suitable for active food packaging, for example antioxidant behavior and antimicrobial activity [3,7,10]. Thus, owing to functionalities of chitosan, the ChNF–CNF composite can be an active food packaging material. 

To evaluate the morphology of CNF and ChNF, several techniques are available, for example scanning electron microscope (SEM), atomic force microscope (AFM), transmission electron microscope (TEM) and particle size analyzer. The size distributions of CNF and ChNF are very broad depending on the isolation methods. ChNF prepared by electrospinning exhibited a diameter ranging from 70 to 330 nm [18]; 260 nm with beads [29]; from 128 to 153 nm without beads [17]; and between 3 nm and few microns, at which the large diameter of nanofiber was due to self-assembly of ChNF [30]. The ChNF produced by grinder and high-pressure homogenizer yielded around 88 nm in diameter [31]. When chitosan nanoparticles were prepared by dissolving then slowly precipitating them in sodium tripolyphosphate solution, the chitosan nanoparticles exhibited a diameter of around 164 nm [32]. In the case of CNF, the TEMPO-oxidized CNF exhibited its width between 3 nm and few microns in length [25]. Its morphology was also investigated by AFM and, after centrifugal fractionation, the average width of the CNF was reduced to 2.0 ± 0.6 nm [33]. Depending on the treatment conditions, not only the size distribution, but also the physical properties including the thermal properties and crystallinity index can be varied. To the best of our knowledge, there has not been any research focused on the conversion of chitosan to ChNF by using ACC method. Furthermore, no research has been attempted to explore ChNF and CNF composites applicable for active food packaging. ChNF–CNF composites can be easily prepared just by blending ChNF and CNF. 

Therefore, in this paper, we investigated the effect of ACC treatment conditions on the properties of ChNF, and prepared ChNF–CNF composites by simply blending two nanofibers, which can be applicable for an active food packaging material. The prepared ChNF–CNF composites as well as ChNF were characterized in terms of morphology, hygroscopic behavior and chemical interaction, as well as thermal, optical, mechanical and antioxidant properties. 

## 2. Materials and Methods 

### 2.1. Materials

Low molecular weight Chitosan was purchased from Sigma Aldrich. The chitosan samples were dipped in deionized (DI) water for at least 30 min before subjected to ACC treatment. Softwood bleached kraft pulp was received from Chungnam National University, Daejeon, South Korea. 2,2,6,6-tetramethylpiperidine-1-oxylradical (TEMPO 98%), sodium bromide (NaBr 99%) and hydrochloric acid (HCl 37%) were purchase from Sigma-Aldrich, St. Louis, MO, USA. Sodium hypochlorite solution (NaClO 12%) was purchase from Yakuri Pure Chemicals Co. Ltd. Uji, Japan. Sodium hydroxide anhydrous (NaOH 98%) was purchase from Daejung, South Korea.

### 2.2. Chitosan Nanofiber Preparation

ACC is a water jet system that uses two high-pressure (200 MPa) water jets colliding with each other to produce a high shear force so as to isolate nanofibers from the original suspension [28,34]. ACC is a benign and environmentally friendly isolation method. Thus, ACC was selected for ChNF isolation by using an ACC machine (ACCNAC–100, CNNT, Korea). The nozzle size of two water jets was 160 µm in diameter. Chitosan suspension of 1% concentration was fed to the ACC machine to extract the ChNF. Different passes of chitosan suspension through the ACC machine was done at 10, 15 and 30 passes. The number of pass indicates how many times the suspension goes through the ACC chamber. 

### 2.3. Cellulose Nanofiber Preparation

To isolate CNF, a combination of chemical method, TEMPO-oxidation and the ACC method was adopted to further decrease the size of CNF. Dried bleached softwood kraft pulp was dipped in DI water for at least 30 min followed by disintegration under high speed food mixer for 10 min. The pulp was then subjected to TEMPO-oxidation treatment by using the chemicals: TEMPO 0.013 g/g, NaBr 0.13 g/g and NaClO 12% 4 mL/g-cellulose. The pH 10 was adjusted by addition of 0.1 M NaOH, and the reaction time was set to 90 min. After the reaction, the TEMPO-oxidized cellulose was neutralized with HCl 0.1 M, followed by the addition of methanol to stop the reaction. The TEMPO-oxidized cellulose was washed several times with DI water. 

The TEMPO-oxidized softwood cellulose was first homogenized by using a homogenizer (IKA T25, IKA, Staufen, Germany) for 10 min at 10,000 rpm to strip off any bundled fibers before going to ACC for smooth treatment. The cellulose suspension was passed through the ACC machine for 10 passes. The transparency and morphology of the prepared CNF were determined by using UV spectroscopy, FE-SEM and AFM.

### 2.4. ChNF–CNF Composites Preparation

To prepare the composites, the CNF was used as a matrix and ChNF was blended to reinforce the composites. The blended suspensions were mixed by using the homogenizer (IKA 25) for 10 min at 10,000 rpm. The mixture was then cast on a polycarbonate substrate by using a doctor blade in a clean room and left to dry on air. The weight percent of ChNF was changed to 3%, 5%, 7%, 10%, 15% and 20%, and the composites were named as CTS3, CTS5, CTS7, CTS10, CTS15 and CTS20, respectively. The thickness of the prepared ChNF–CNF composites was between 40 and 45 µm.

### 2.5. Characterizations

#### 2.5.1. Morphology

Morphologies of the prepared ChNF as well as ChNF–CNF composites were investigated by using a field emission scanning electron microscopy (FE-SEM, S-4,000, Hitachi, Japan) and an atomic force microscopy (AFM, Veeco 3100, USA). Since the morphologies of CNF are well reported [21,22,23,24,25], they are not repeated in this paper.

#### 2.5.2. FTIR Spectra

FTIR spectra of the ChNF–CNF composites and ChNF were determined using a FTIR spectroscopy (Cary 630, Agilent Technol. Santa Clara, CA, USA) with a diamond crystal that has the wavelength range from 650 to 4000 cm^−1^. The specimens were tested for the absorbance between 650 and 4000 cm^−1^ with accumulation of 32 scans and the data were collected at a resolution of 4 cm^−1^. 

#### 2.5.3. X-ray Diffraction (XRD)

The crystallinity index (CrI) of ChNF and ChNF–CNF composites were measured using an X-ray diffractometer (XRD, X’Pert PRO MRD, Malvern). It is hard to make chitosan particles into a thin film of 45–70 g/m^2^. Thus, the pure chitosan was used in powder form. A thin ChNF film of 40–45 µm was cast on a polycarbonate substrate by using the doctor blade, and 2 × 2 cm^2^ specimens were prepared to evaluate the CrI. The CrI of chitosan was calculated using the following equation [7].

CrI (%) = (I_002_ − I_am_)/I_002_ ∗ 100 (%) (1)

where I_002_ is XRD peak at 2θ = 19.7° and I_am_ is diffraction pattern of amorphous area at 2θ = 15°. The prepared ChNF–CNF composites were directly used for XRD. 

#### 2.5.4. Mechanical Properties

Tensile test was performed to evaluate the mechanical properties of the prepared ChNF–CNF composites by using a tensile test machine [35]. Specimens were cut to the size of 0.5 × 5 cm^2^. The length between grips was 3 cm. The specimen thickness was varied from 40 to 45 µm. Dried samples were kept in a condition chamber (30% RH and 25 °C) for at least 8 h before the tensile test, and five specimens were tested for each case. The ChNF specimen was prepared by casting it and tested also for comparison. 

#### 2.5.5. Thermogravimetric Analysis

Thermal stability of the prepared ChNF and ChNF–CNF composites were analyzed by using Thermogravimetric analyzer (TGA, STA 409PC, NETZSCH, Selb, Germany). Seven milligrams of the sample were prepared and thermally induced starting from 30 °C until 500 °C.

#### 2.5.6. Viscosity 

The viscosity of the prepared ChNF suspension was investigated by using a viscometer (LV DV2T, Brookfield viscometer, USA). The spindle LV–04 (64), speed 0.1 rpm, T = 23.5 °C and time recorded from 1 to 5 min were chosen for the test condition, and 0.8 wt% of ChNF suspensions were provided. The ChNF yield was investigated by using centrifugation at 7000 rpm for 1 h. 

#### 2.5.7. UV–Transmittance

The UV transmittance of the prepared ChNF and ChNF–CNF composites were investigated by using a UV spectrometer (HP 845×, Hewlett-Packard, Hayward, CA, USA). Suspensions of the specimens were used to measure the UV transmittance at the wavelength range of 200–800 nm.

#### 2.5.8. Hygroscopic Behaviors

Water contact angle (WCA) measurement of the pure CNF and ChNF–CNF composites was carried out. A drop of 5 µL was deposited on a thin film and the images were taken by AMcap software and then analyzed using ImageJ tool. Water vapor transmittance rate (WVTR) was tested according to ASTM standard E 96-95 [36]. The sample was kept in a humidity chamber at 25 °C and 50% RH. The WVTR was taken hourly for up to 8 h continuously

#### 2.5.9. Antioxidant Property 

The antioxidant activity of the prepared ChNF–CNF composites was tested by using ABTS free radical [37]. The antioxidant activity analysis was based on the discolored radicals of ATBS after 40 min under UV light measurement at 734 nm. The UV absorption was adjusted to absorption at 0.8; 20 mg of CNF, ChNF and ChNF–CNF composites in 2 mL of ABTS were used for the measurement after 40 min. In other words, various chitosan contents (at 0.03, 0.05, 0.07, 0.1, 0.15 and 0.2 mg/mL of ABTS) were used. All antiradical tests were carried out twice for each sample. Then, 7 mM ABTS was dissolved in DI water and mixed with 2.45 mM potassium persulfate and kept in a dark drawer for 16 h. After that, the ABTS suspension was diluted with methanol to adjust the absorption of 0.8 at wavelength 734 nm. The antioxidant activity of CNF and ChNF–CNF composites was calculated by the following equation:

AO(%) = 100 ∗ (1−A_a_/A_o_) (2)

where AO is the antiradical activity, A_o_ is the absorption of the control ABTS solution and A_a_ is the absorption of the ABTS solution with sample in steady state.

## 3. Results

### 3.1. Chitosan Nanofibers

**Figure 1:**
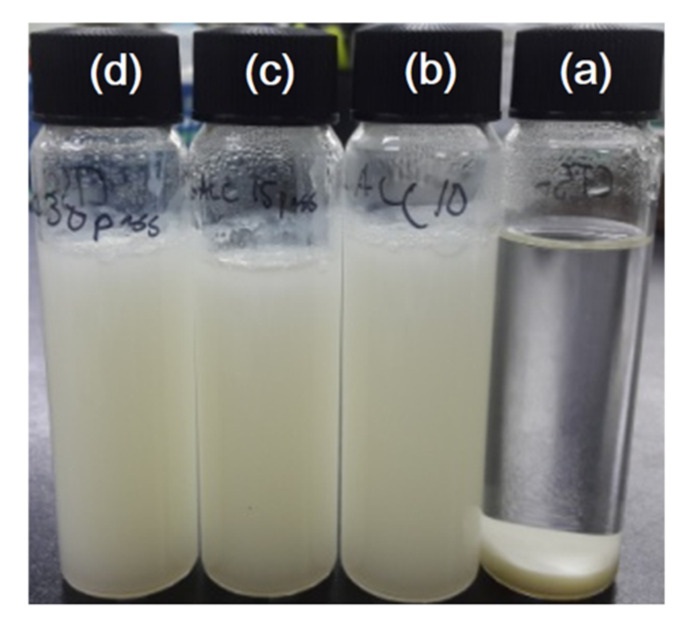
Photograph of ChNF suspensions: (**a**) the original chitosan; (**b**) after 10 ACC passes; (**c**) after 15 ACC passes; and (**d**) after 30 ACC passes.

**Figure 2:**
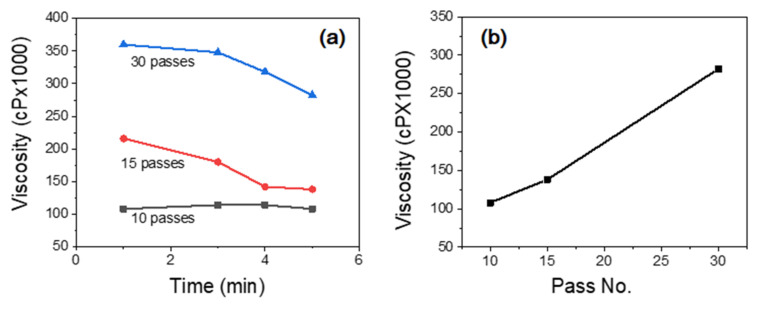
Effect of ACC passes on the viscosity of ChNF suspension: (**a**) viscosity change with time, and (**b**) viscosity change with ACC pass number.

Figure 1 shows the photograph of ChNF suspensions treated with 10, 15 and 30 passes of the ACC treatment. After ACC treatment, the suspensions were changed to ivory color, and as the ACC pass increased the suspension color turned to a milky. The viscosity of ChNF suspensions was measured and Figure 2 shows the result. The viscosity of ChNF suspensions increased with the number of ACC pass. The higher is the number of passes at the ACC chamber, the higher is the viscosity of ChNF. Higher viscosity means a higher fibrillation of chitosan, thus reducing its size to nanofibers. Note that the low viscosity ChNF samples (10 and 15 ACC passes) easily flowed when the samples were placed upside down, while the 30 passes case did not flow when it was placed upside down. Interestingly, the viscosity values decreased with the time especially, for the 15 and 30 passes cases. This might be associated with the broken inner bonds in ChNF and the layer separation in the suspension. Figure 2b shows the viscosity change with the number of ACC pass when the time is 5 min. The viscosity linearly increased with the number of ACC pass. 

The yield of ChNF was evaluated in each sample by centrifugation. The result shows that, after 10, 15 and 30 passes of the ACC treatment, the yields of ChNF were 13.2%, 15.5% and 27.7%, respectively. From the viscosity and yield data, it is clear that the higher is the number of ACC passes, the better is the isolation of ChNF. 

**Figure 3:**
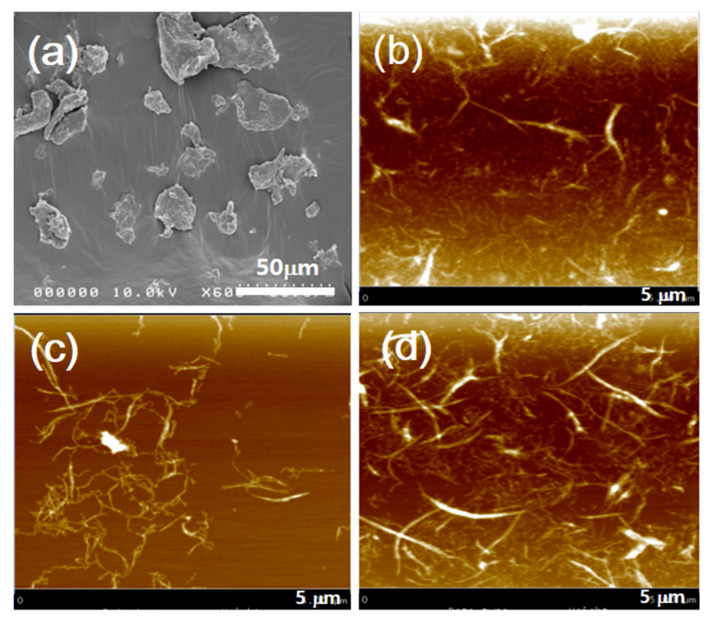
Morphologies of chitosan and its nanofibers: (**a**) SEM image of the original chitosan particles; and AFM images of ChNFs with different ACC passes: (**b**) 10 passes; (**c**) 15 passes; and (**d**) 30 passes (AFM images are in 5 μm × 5 μm).

The morphology changes of chitosan after the ACC treatment were observed by FE-SEM and AFM. Figure 3a–d shows the morphologies of the original chitosan and ChNFs with 10, 15 and 30 passes of the ACC treatment, respectively. The chitosan particles were around 50–100 µm. After the ACC treatment, it was changed to nanofibers, as shown in Figure 3b–d. After 30 passes, the ChNF size was reduced to 38 ± 16.5 nm in width and length of several microns. The width of ChNF was calculated in 100 measurements from the AFM images. This size of ChNF is larger and longer than that of CNF. Thus, in the preparation of ChNF–CNF composites, 30 passed ChNF was chosen.

### 3.2. ChNF–CNF Composites

#### 3.2.1. Morphologies

**Figure 4:**
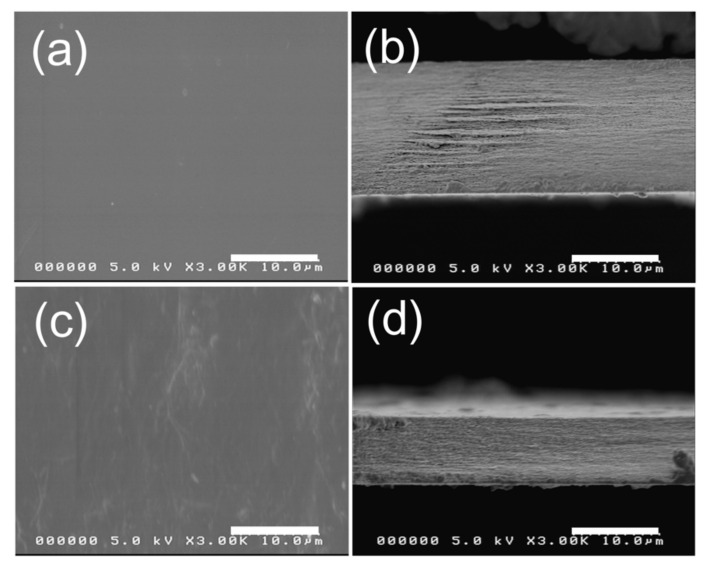
FE-SEM images of: (**a**) surface of CNF film; (**b**) cross section of CNF film; (**c**) surface of ChNF–CNF composite; and (**d**) cross section of ChNF–CNF composite.

The morphologies of the ChNF–CNF composite structure were investigated. Figure 4 shows the SEM images of the ChNF–CNF composite and CNF film. Figure 4a shows a smooth surface of the CNF film and Figure 4b is the cross-section SEM image of the CNF film, which exhibits the layer-by-layer structure of CNF. After blending with ChNF, the surface of ChNF–CNF composite turned out to be rough. The cross-section SEM image of the composite also shows a layer-by-layer structure similar to the CNF film. It was shown that the ChNF was well blended with CNF to maintain the layer-by-layer structure. 

**Figure 5:**
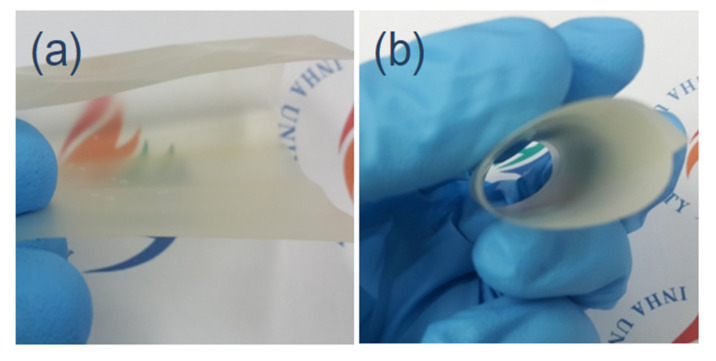
Flexibility of ChNF–CNF composite by: (**a**) bending; and (**b**) rolling.

In addition, to consider ChNF–CNF composites for packaging, the composites should be bendable. Thus, a quick bending test was performed with the ChNF–CNF composite. The composites were so flexible that they could be bent in any direction without cracking. The thickness of the composites was in the range of 40–50 µm. Figure 5 shows flexibility of the composite in bending and rolling deformation.

#### 3.2.2. FTIR

**Figure 6:**
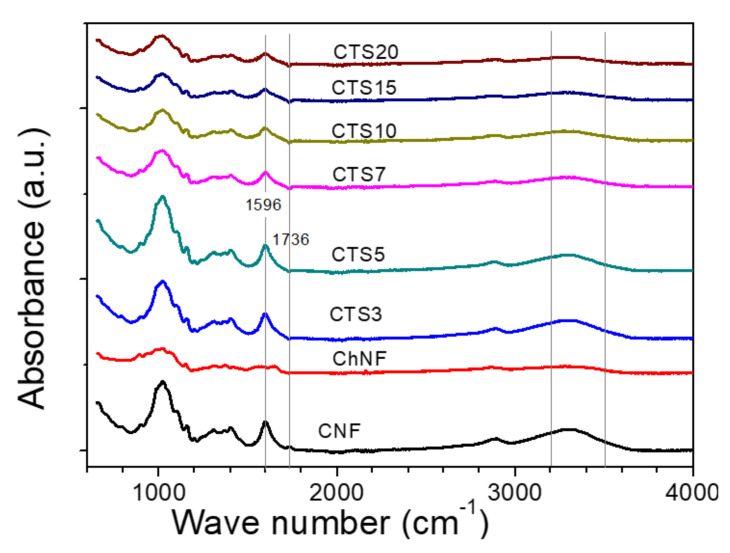
FTIR of CNF, ChNF and ChNF–CNF composites.

Figure 6 shows the FTIR spectra of the CNF, ChNF and ChNF–CNF composites with different ChNF concentration. The peak in the range of 3200–3500 cm^−1^ of the ChNF–CNF composites was assigned to the hydrogen-bonded O–H stretching in both CNF and ChNF. Chitosan showed broader O–H bonded as compared to cellulose with higher intensity. Due to the combination of these two materials, with 20% chitosan, the intensity of the peak at 3200–3500 cm^−1^ appeared similar to pure chitosan. The CNF showed stronger and higher peak of hydrogen-bonding than the ChNF. When ChNF and CNF were blended, peaks at 3000 and 3500 cm^−1^ were reduced, which might be due to the intra-bonding between two materials. The peak at 1596 cm^−1^ assigned for –C=O stretching is clear for CNF. The peak at 1736 cm^−1^ was assigned for the C=O stretch of the –COOH group. This was only observed in the CNF at first, and the intensity of this peak decreased due to the addition of ChNF. At last, this peak became weak and disappeared when the ChNF concentration increased. This phenomenon indicates the interaction between ChNF and CNF. The peak at 1596 cm^−1^ only appeared for cellulose and composites but not clearly shown for chitosan. This peak might be associated with the aromatic ring stretching of lignin or hemicellulose remained small amount in the bleached kraft pulp. From the FT-IR analysis, the structure of CNF and ChNFs were confirmed in the composites. 

#### 3.2.3. Crystallinity Index 

**Figure 7:**
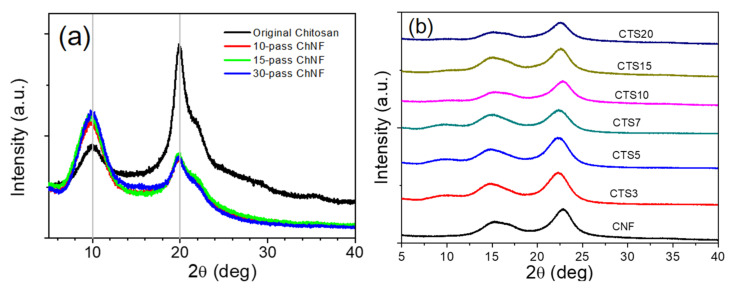
Crystallinity index of: (**a**) ChNF with number of ACC passes; and (**b**) CNF and ChNF–CNF composites.

The crystallinity index (CrI) of the original chitosan and ChNF were obtained by XRD and calculated according to Equation 1 as shown in Figure 7a. The crystalline peaks of chitosan appeared at 9.7° and 19.7°. The peak at 19.7° decreased by the ACC treatment, while the strong peak at 9.7° increased, which might be associated with the incorporation of bound water molecules of α-chitin [38]. It means that a higher ACC treatment leads to more water molecules bound on hydrophilic surface of ChNF. The CrI values of the original chitosan and ChNFs with 10, 15 and 30 ACC passes were shown to be 65.4%, 44.0%, 44.0% and 36.2%, respectively. The CrI of chitosan highly degraded after ACC treatment. Note that 30 ACC passed ChNF was used for the ChNF–CNF composite preparation. 

The XRD patterns of the ChNF–CNF composites are shown in Figure 7b with different ACC passes. As one can see, cellulose peaks at around 15.8° and 22.7° were clearly shown and as the ChNF content increased, chitosan peaks were slightly appeared by interfering with cellulose peaks. Thus, CrI of the composites was calculated mainly by the dominant cellulose peaks at 22.7°. The CrI values for low ChNF content composites slight improved at low chitosan content (ChNF3 and ChNF5) from 63% to 65%, and then it slightly decreased to 58% with addition of ChNF content. This might be due to good miscibility between ChNF and CNF. 

#### 3.2.4. Mechanical Properties

Tensile test was performed for ChNF, CNF and ChNF–CNF composites and the results are shown in Table 1. The tensile strength of the composite at first was 174.5 MPa (no ChNF), reached 224.0 MPa when the ChNF content was 10% and then decreased thereafter. Note that mechanical properties of the pristine ChNF is much lower than the CNF, i.e. more flexible than the CNF. Similarly, the yield strength of the composite was initially 111.7 MPa and reached its maximum of 149.0 MPa when the ChNF content was 10%. It was shown that the elongation at break of the composite increased from 2.02% to 4.17% and then decreased. It was shown that, when reinforced by ChNF, the tensile strength, yield strength and elongation at break of the composite improved. However, Young’s modulus of the composite was not improved and slightly decreased due to the low modulus of ChNF. The modulus of ChNF is 7.3 GPa, while that of CNF is 16.9 GPa.

**Figure 8:**
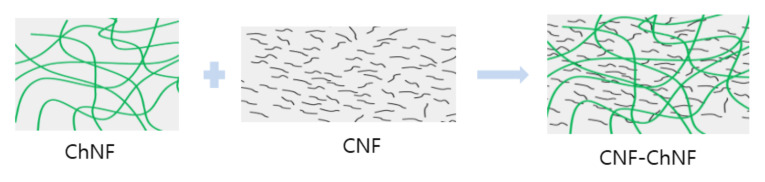
A possible reinforcement mechanism of ChNF and CNF composite.

Nevertheless, the overall mechanical properties of the composite were improved, which might be due to the reinforcement of ChNF to CNF in the composite. Most previous studies produced cellulose–chitosan composites by dissolving chitosan or cellulose and blending them [2,32]. However, in this research, we adopted the non-dissolving method to prepare ChNF–CNF composites, and a remarkable improvement of the mechanical properties of the ChNF–CNF composite was observed. There could be several mechanisms that can explain the mechanical properties improvement of the composite. The first one is improvement of the bonding sites between ChNF and CNF due to different size of ChNF and CNF. Note that the ChNF size was larger than that of CNF. The long length of ChNF in the CNF matrix and high surface areas of CNF created many reaction sites and bonding areas on their surfaces. Furthermore, the CNF is relatively stiff and ChNF is flexible. The mismatched mechanical properties of ChNF and CNF could give a room for managing the composite mechanical properties. Figure 8 shows the possible concept of reinforcement in the composite. By blending these two heterogeneous polysaccharide nanofibers, we can manage the mechanical properties of the composite. It is hoped that this idea could be a way to produce cellulose–chitosan composites with better mechanical properties without dissolving process of chitosan or cellulose. 

#### 3.2.5. Thermal Stability

**Figure 9:**
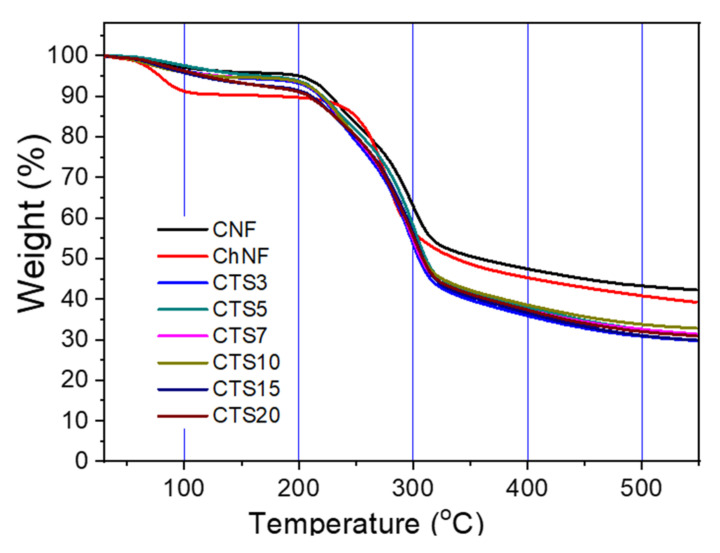
TGA of ChNF–CNF composites.

The thermal stability of ChNF, CNF and their composites were examined by TGA, and the results are shown in Figure 9. At 30 to 100 °C of the evaporation stage, there was no significant difference in the first stage of water evaporation in the composites except ChNF. The CNF and their composites started to degrade around 200 °C and reached around 320 °C for the first phase of degradation, while ChNF started to degrade near 250 °C. The composites showed almost the same thermal degradation range as the CNF. It can be seen that they are stable up to 250 °C. Note that the CNF and ChNF exhibited larger residuals than the composites. The residuals after 320 °C was around 40–50% for CNF and ChNF. The reason for larger residuals of the CNF and ChNF than the composites is under investigation. 

#### 3.2.6. UV Transmittance 

**Figure 10:**
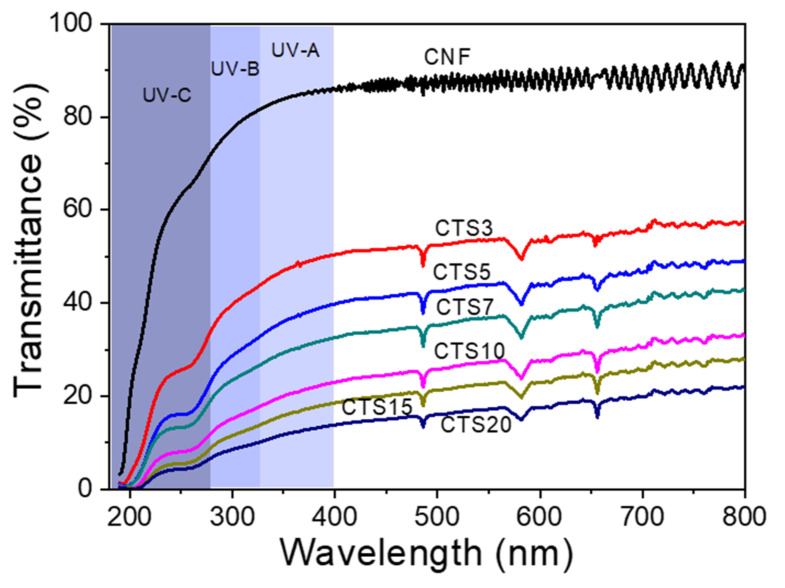
UV-transmittance of ChNF–CNF composites.

UV protection of ChNF–CNF composites is an important factor for food packaging applications. UV transmission of the composites was evaluated, and the results are shown in Figure 10. As compared to the neat CNF, the ChNF–CNF composites showed better UV-protection. Higher CTS content showed better the UV protection. In the range of UV-A, UV-B, and UV-C, CTS10, CTS15 and CTS20 exhibited higher UV protection than CTS3, CTS5 and CTS7. However, transparency of the composites is sacrificed to improve the UV protection. Thus, good UV protection with maintaining good transparency is future work. 

#### 3.2.7. Hygroscopic Behaviors

WCA and WVTR were tested to investigate the hygroscopic behaviors of the composites. Table 2 shows the results. The WVTR was a little increased at first by the addition of ChNF up to 10% and then saturated thereafter. This result shows a good agreement with the previous research for CNF and acetylated CNF [22]. Regarding the WCA of the composites, it was rather a bit decreased by adding ChNF, which indicates hydrophilic behavior of the composites. This hydrophilic behavior might be associated with the hydroxyl groups appeared on the surface of the composites. Enhancing this behavior is future work in this research. 

#### 3.2.8. Antioxidant Property

**Figure 11:**
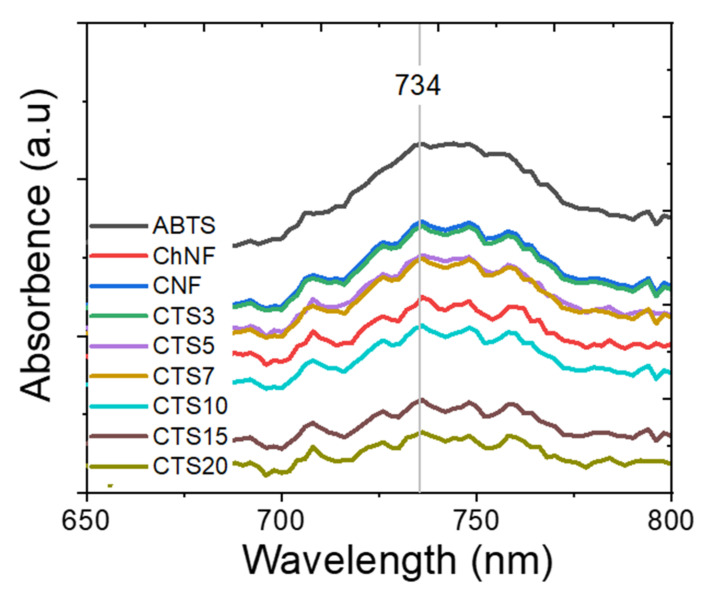
Antioxidant of ChNF–CNF composites.

The antioxidant abilities of CNF, ChNF and ChNF–CNF composites were investigated and expressed in AOA%/100 mg, as shown in Figure 11. The antioxidant activities of CNF, ChNF, CTS3, CTS5, CTS7, CTS10, CTS15 and CTS20 were 16.9%, 27.9%, 19.4%, 23.4%, 23.4%, 33.4%, 47.4% and 52.0%, respectively. The CTS20 showed the highest antioxidant activity among all cases. Interestingly, although the CNF and ChNF have low antioxidant activities, when they were blended together, the antioxidant activity of the composites increased. Cellulose and chitosan are both polysaccharides, and, when they are combined, the reducing ends of the materials may open, thus increasing the antioxidant activity. Nanofibrillation can also increase the hydroxyl groups, which are good for antioxidant activities. The antioxidant activity is the interaction of free radicals with the hydroxyl groups, and free amino groups of the chitosan and cellulose [39,40]. The improvement of antioxidant activities of the composites is agreeable with the previous research [39], which showed a steady increase of the antioxidant activity of the composites by increasing the chitosan content. 

## 4. Conclusions

In this research, ChNF was isolated by using the ACC method and increasing the number of ACC passes reduced the size and increased the yield of ChNF. Isolating CNF by using the combination of TEMPO oxidation and the ACC method resulted in smaller size than that of ChNF. By blending two heterogeneous polysaccharide nanofibers, we were able to prepare ChNF–CNF composites. ChNF was well blended with CNF so as to maintain the layer-by-layer structure of the composites. The composites were so flexible that they could be bent in any direction without cracking. From the FT-IR analysis, the structure of CNF and ChNFs were confirmed in the composites. The composites showed remarkable improvement of the mechanical properties, which might be due to the reinforcement of ChNF to CNF in the composites. The composites showed almost the same thermal degradation range of the CNF. Better UV-light protection was shown at higher content of ChNF in the composites. The WVTR was slightly increased at first by the addition of ChNF up to 10% and then saturated thereafter. The WCA of the composites was rather slightly decreased by adding ChNF, and enhancing this behavior is future work. The high ChNF content showed the highest antioxidant activity in the composites. Although the CNF and ChNF had low antioxidant activities, their blended composites had increased the antioxidant activity. It is the first time a simple combination of ChNF–CNF composites fabrication showed good mechanical properties and antioxidant activities. Reinforcement of ChNF into CNF was a good selection in terms of antioxidant activity, UV protection and mechanical properties. The ChNF–CNF composite could be useful for active food packaging application. 

## Figures and Tables

**Table 1:** Tensile test results of ChNF, CNF and their composites.

Samples	Young’s Modulus (GPa)	Yield Strength (MPa)	Tensile Strength (MPa)	Elongation at Break (%)
CNF	16.9 + 2.6	111.7 + 57	174.5 + 56.0	2.02
CTS3	13.9 + 2.1	100.2 + 23	183.1 + 60.8	2.06
CTS5	15.2 + 4.0	124.6 + 17	197.5 + 32.0	2.46
CTS7	13.2 + 0.5	136.2 + 80	216.5 + 95.7	2.85
CTS10	12.9 + 0.9	149.0 + 20	224.0 + 27.0	4.17
CTS15	13.5 + 2.3	131.8 + 23	198.0 + 31.4	3.40
CTS20	15.8 + 2.7	104.4 + 22	157.1 + 30.5	1.79
ChNF *	7.3 + 0.70	87.5 + 26.8	133.4 + 17.9	3.69

* when number of ACC passes is 30.

**Table 2:** Water vapor transmission rate and water contact angle of CNF and ChNF–CNF composites.

Sample	Water Vapor Transmission Rate (g /m^2^.day)	Water Contact Angle (°)
CNF	164.98 + 3.09	47
CTS3	164.69 + 1.85	47
CTS5	167.33 + 5.32	45
CTS7	171.18 + 0.89	45
CTS10	175.29 + 1.08	41
CTS15	173.45 + 0.67	41
CTS20	173.04 + 6.06	38
